# Higher β cell death in pregnant women, measured by DNA methylation patterns of cell-free DNA, compared to new-onset type 1 and type 2 diabetes subjects: a cross-sectional study

**DOI:** 10.1186/s13098-023-01096-9

**Published:** 2023-06-01

**Authors:** Teresa María Linares-Pineda, Carolina Gutiérrez-Repiso, Nerea Peña-Montero, María Molina-Vega, Fuensanta Lima Rubio, María Suárez Arana, Francisco J. Tinahones, María José Picón-César, Sonsoles Morcillo

**Affiliations:** 1 Unidad de Gestión Clínica de Endocrinología y Nutrición del Hospital Virgen de la Victoria, Instituto de Investigación Biomédica de Málaga (IBIMA), Málaga, Spain; 2 Centro de Investigación Biomédica en Red de Fisiopatología de la Obesidad y la Nutrición (CIBERobn), Instituto de Salud Carlos III, Madrid, Spain; 3 Department of Obstetrics and Gynecology, Hospital Regional Universitario de Málaga, IBIMA, Málaga, Spain; 4 Departamento de Medicina y Dermatología, Universidad de Málaga, Málaga, Spain

**Keywords:** Cell-free DNA, Biomarker, Gestational diabetes mellitus (GDM), Type 2 diabetes mellitus (T2DM), Type 1 diabetes mellitus (T1DM), β cell death, DNA methylation

## Abstract

Diabetes is a metabolic disorder of glucose homeostasis in which β cell destruction occurs silently and is detected mainly when symptoms appear. In the last few years, it has emerged a great interest in developing markers capable of detecting pancreatic β cell death focused on improving early diagnosis and getting a better treatment response, mainly in type 1 diabetes. But other types of diabetes would also benefit from early detection of β cell death. Differentially methylated circulating DNA is being studied as minimally invasive biomarker of cell death. We aimed to explore whether the unmethylated/methylated ratio of the insulin and amylin genes might be a good biomarker of β cell death in different types of diabetes. A lower index ∆Ct indicates a higher rate of β-cell death. Plasma samples from subjects without diabetes, pregnant women, pregnant with gestational diabetes (GDM), type 1 diabetes and type 2 diabetes were analyzed. A qPCR reaction with specific primers for both methylated and unmethylated fragments of insulin and amylin genes were carried out. Pregnant women, GDM and non- GDM, showed a higher β-cell death for both markers (∆INS = 3.8 ± 2.1 and ∆Amylin = 8.5 ± 3.6), whereas T1D presented lower rate (∆INS = 6.2 ± 2.1 and ∆Amylin = 10.7 ± 2.9) comparable to healthy subjects. The insulin methylation index was associated with the newborn birth weight (r = 0.46; p = 0.033) and with insulin resistance (r = -0.533; p = 0.027) in the GDM group. The higher rate of β-cell death was observed in pregnant women independently of their metabolic status. These indexes could be a good indicator of β cell death in processes caused by defects on insulin secretion, insulin action, or both.

**Supplementary Information:**

The online version contains supplementary material available at 10.1186/s13098-023-01096-9.

## Introduction

Diabetes mellitus (DM) is a disorder of glucose homeostasis, characterized by elevated levels of blood glucose, which leads over time to serious damage to the heart, blood vessels, eyes, kidneys, and nerves. In general, DM is caused by defects on insulin secretion, insulin action, or both. Insulin- producing β cells in the pancreas are responsible for the proper maintenance of glucose homeostasis. The loss of β cells is considered a key to the pathogenesis of both type 1 diabetes (T1D) and type 2 diabetes (T2D) [1]. In the case of T1D, β cells are destroyed via T-cell-mediated immunity. This is a silent process that can be detected only when a high percentage (60–70%) of β cell mass has been lost [2]. Although T2D results from the combination of resistance to insulin action and inadequate insulin secretion, β cell death also occurs in the progression of the disease [3]. The same as T1D, the clinical manifestation of disease takes place after a substantial loss of functional β cell mass. In pregnancy, many metabolic changes such as a reduction in insulin sensitivity are produced to ensure nutrients supply to the fetus. Consequently, adaptations in β-cell function have been observed to avoid developing hyperglycemia. When adaptation fails, Gestational Diabetes Mellitus (GDM) occurs. GDM is characterized by insulin resistance and impaired β cell function, but less is known about the role of β cell death in this stage [4]. In the last few years, it has emerged a great interest in developing markers capable of detecting pancreatic β cell death focused on improving early diagnosis and getting a better treatment response, mainly in type 1 diabetes. Traditional biomarkers such as C-peptide and islet autoimmune antibodies have some limitations to detect β cell death. The C-peptide levels reflect the residual β cell function rather than beta cell death. In addition, c-peptide is easily affected by other factors such as insulin resistance. Regarding islet autoimmune antibodies, they have limited sensitivity and discriminate just a tiny part of people who will finally develop T1D. Not all the patients with a positive islet autoimmune antibodies ended up developing T1D [5]. So, recently, a new biomarker has been proposed as a good candidate reflecting β cell death. Several authors have detected β cell death using differentially methylated circulating DNA [6–11]. This method is based on the specific methylation pattern of each cell type. Although the nucleotide sequence is identical for each cell, the methylation profile is tissue specific and unique to each cell type [12]. Some researchers have identified unique unmethylated CpGs sites of β cells, in Insulin, Amylin, Glucokinase and CHTOP genes [13–16]. Cell free DNA (cfDNA) are nucleic acid fragments that enter the bloodstream during apoptosis or necrosis and contain signals of the tissue from which they are derived [17]. Therefore, the detection of differentially methylated specific CpGs sites in serum or plasma sample could be a good biomarker of cell death. In the case of β cell death, most studies have focused on the Insulin gene (*INS*) which is expressed almost exclusively in islet β cells [6]. The *INS* gene presents a specific pattern of unmethylated CpGs sites in coding and non-coding regions, whereas these cytosines are methylated in non-β cell types. The increasing interest of these potential biomarkers has been driven by the idea that non-symptomatic T1D subjects could be identified even prior to the appearance of autoantibodies [18]. Regarding other genes, only one study has explored the Islet Amyloid Polypeptide (IAPP), also known as amylin, in recent onset T1D patients. Other research carried out by Sklenarova et al. analyzed the presence of unmethylated cfDNA from GCK gene in samples from children with recent-onset T1D and first-degree relatives of T1D patients, suggesting that this gene could be more suitable than Insulin gene for detection of β cell death [15]. Recently, Mirmira et al., identified another gene (CHTOP) by unbiased DNA methylation analysis using data from the Infinium Human Methylation 450 array [16]. This new assay was performed in samples from T1D, T1D/first-degree relative, lean and overweight/obese youth.

To date, these assays have been developed using a variety of technology, with different handling and processing prior to cfDNA extraction, and distinct cohorts. Most of the studies have been focused on *INS* gene, however other markers have been proposed but they have not been corroborated in other studies. Whilst the majority of the studies have been directed to detect β cell death mainly in subjects with T1D or in subjects at risk for T1D, much less is known in other types of diabetes mellitus or carbohydrate metabolism disorders.

The objective of this study was to evaluate the capacity of detection of β-cell death by two markers based on differentially methylated pattern of cfDNA (*INS* and Amylin genes) in different type of diabetes mellitus.

## Methods

### Subjects

Plasma samples were collected from subjects without diabetes (control group, N = 10), pregnant women (non-GDM, n = 25), pregnant with gestational diabetes (GDM, n = 25), type 1 diabetes (T1DM, n = 16) and type 2 diabetes (T2DM, n = 18).

Pregnant women, referred to the Diabetes and Pregnancy Unit after a positive O'Sullivan test, were diagnosed with gestational diabetes by oral glucose overload (100 gr- OGTT) at 24–30 weeks of pregnancy according to National Diabetes Data Group NDDG [19]. Following the result of the OGTT-100 g patients were classified as GDM or non-GDM.

Subjects who attended to the Diabetes Day Hospital with clinical onset of diabetes were diagnosed as T1DM or T2DM according to c-peptide levels, HbA1c levels and presence of autoantibodies GAD e IA2 by ADA criteria [20].

All participants gave their consent to participate in the study. The study was approved by the Institutional review board at the Hospital University Virgen de la Victoria of Málaga, Spain.

### Procedures

Plasma was obtained by whole blood centrifugation at 4000*g* for 15 min at 4 ºC. Supernatant was separated and centrifuged again at 16000*g* for 10 min at 4 ºC. All the samples were stored at -80ºC until posterior isolation of cell free DNA (cfDNA). cfDNA was extracted from 1,5–2 ml of plasma using Qiamp MinElute ccfDNA Mini Kit (Qiagen GmbH., Hilden, Germany) following the manufacturer´s Instructions. Quantification and integrity of cfDNA was performed with High sensitivity DNA ScreenTape Analysis and TapeStation Systems (Agilent Technologies., Waldbronn, Germany). Concentrations were, in all the cases, lower than 800 pg/µl. Finally, a volume of 20 µl of cfDNA was treated with bisulfite by Epitect Bisulfite Kit (Qiagen GmbH., Hilden, Germany), following the protocol for DNA from Low Concentration Solutions, from the manufacturer.

Serum insulin was measured by immunoassays using Atellica IM Insulin (Siemens Healthcare Diagnostics, Spain). HOMA-IR was calculated by the formula: fasting insulin (microU/L) x fasting glucose (nmol/L)/22.5 [21].

### Β-cell death marker assays

Based on previously published data we carried out two assays. Most studied CpGs sites from INS gene were selected. The assay was addressed to the positions + 396 and + 399 of the human Insulin gene (*INS*) [22]. Regarding Amylin gene, only one study has been carried out. This assay focused on the positions + 5414 and + 5419 of the Amylin gene (Amylin or IAPP) [14]. In both cases a nested PCR was performed. A first independent-methylation PCR reaction was carried out to increase the cfDNA template. This PCR product was used in a qPCR reaction with specific primers for both methylated and unmethylated fragments of INS and Amylin genes. The reaction was run in a QuantSudioTM Pro Real-Time 6 PCR (Applied Biosystems, ThermoFisher Scientific, Waltham, MA, USA) and the *C*_*t*_ value for methylated and non-methylated fragments were obtained. *C*_*t*_ value indicates the number of cycles in which fluorescence increases significantly. The PCR conditions and primers are shown in Additional file 1: Table S1.

The relative abundance of unmethylated DNA was expressed as the difference (∆) of *C*_*t*_ values between unmethylated and methylated fragments as previously published by Lebastchi [23]. Considering that high values of *C*_*t*_ signify lower amount of cfDNA, a lower difference (∆*C*_*t*_: *C*_*t*_ unmethylated-*C*_*t*_ methylated) indicates a higher proportion of unmethylated DNA, therefore a higher rate of β-cell death.

### Statistical analysis

Data are expressed as mean ± standard deviation, or percentages. Differences between groups were analyzed by one-way Anova test. Post hoc analysis was done by Duncan test. Proportions were compared using Chi-Square test and quantitative variables by correlation Spearman test. Statistical analyses were performed using R 3.5.1 (https://www.r-project.org) and graphics were generated by Graph Pad software (https://www.graphpad.com/). Statistical significance was considered when P value < 0.05.

## Results

### Characteristics of the study subjects

Four groups of subjects were studied: subjects without diabetes (control), pregnant women diagnosed with gestational diabetes (GDM), pregnant women without gestational diabetes (non-GDM), and finally a group of subjects who debuted with diabetes mellitus and were diagnosed as Type 1 Diabetes (T1D) and Type 2 Diabetes (T2D). The main characteristics of these subjects are shown in Table 1. Age, weight, and glucose levels were significantly different between groups. Subjects who attended to the Diabetes Day Hospital with the clinical onset of diabetes were diagnosed as type 1 diabetes or type 2 diabetes according to c-peptide levels, HBA1c levels and presence of autoantibodies GAD e IA2. Subjects with T1D were younger and thinner than T2D patients.

**Table 1:** Characteristics of the subjects

	Control N = 10	GDM N = 25	NON-GDM N = 25	T1D N = 16	T2D N = 18	p
Age(years)	36.30 ± 7.7^a^	35.16 ± 4.01^a^	33.19 ± 4.38^a^	30.44 ± 14.21^a^	44.94 ± 13.21^b^	< 0.001
Sex(%)(M/F)	23/77	0/100	0/100	31.3/68.7	76.5/23.5	NA
Weight (Kg)	64.1 ± 13.5^a.b^	79.61 ± 15.7^c^	75.3 ± 17.3^b.c^	57.7 ± 11.1^a^	94.98 ± 20.23^d^	< 0.001
Fasting glucose (mg/dl)^*^	83.87 ± 9.9^a^	84.2 ± 10.9^a^	83.46 ± 7.64^a^	252 ± 107.3^b^	264.12 ± 83.41^b^	< 0.001
BMI	22.7 ± 3.9^a^	30.1 ± 5.1^b^	30.1 ± 5.4^b^	21.5 ± 5.0^a^	31.9 ± 7.2^b^	< 0.001
HbA1c	–	5.3 ± 0.3^a^	5.1 ± 0.3^a^	13.7 ± 4.4^b^	14.0 ± 4.7^b^	
c-Peptide	–	4.2 ± 4.5^a^	1.4 ± 0.3^b^	0.52 ± 0.3^b^	1.6 ± 1.0^b^	
GAD positive (%)				56.3	0	< 0.001
IA2 positive(n/%)				43.8	0	< 0.001
Newborn weight (gr)		3.097 ± 0.345	3.277 ± 0.382			NS
HOMA-IR		2.9 ± 2.3	1.6 ± 0.9			0.045

Data are expressed as mean ± SD or percentages. Mean values were compared by one way ANOVA test. Post hoc analysis was done by Duncan test. Different letters indicate statistically significant differences between groups. *Fasting glucose was measured for all the participants except for T1D and T2D patients who attended to the clinic without an appointment

### Levels of β-cell derived cfDNA in the different groups of subjects

**Fig. 1:**
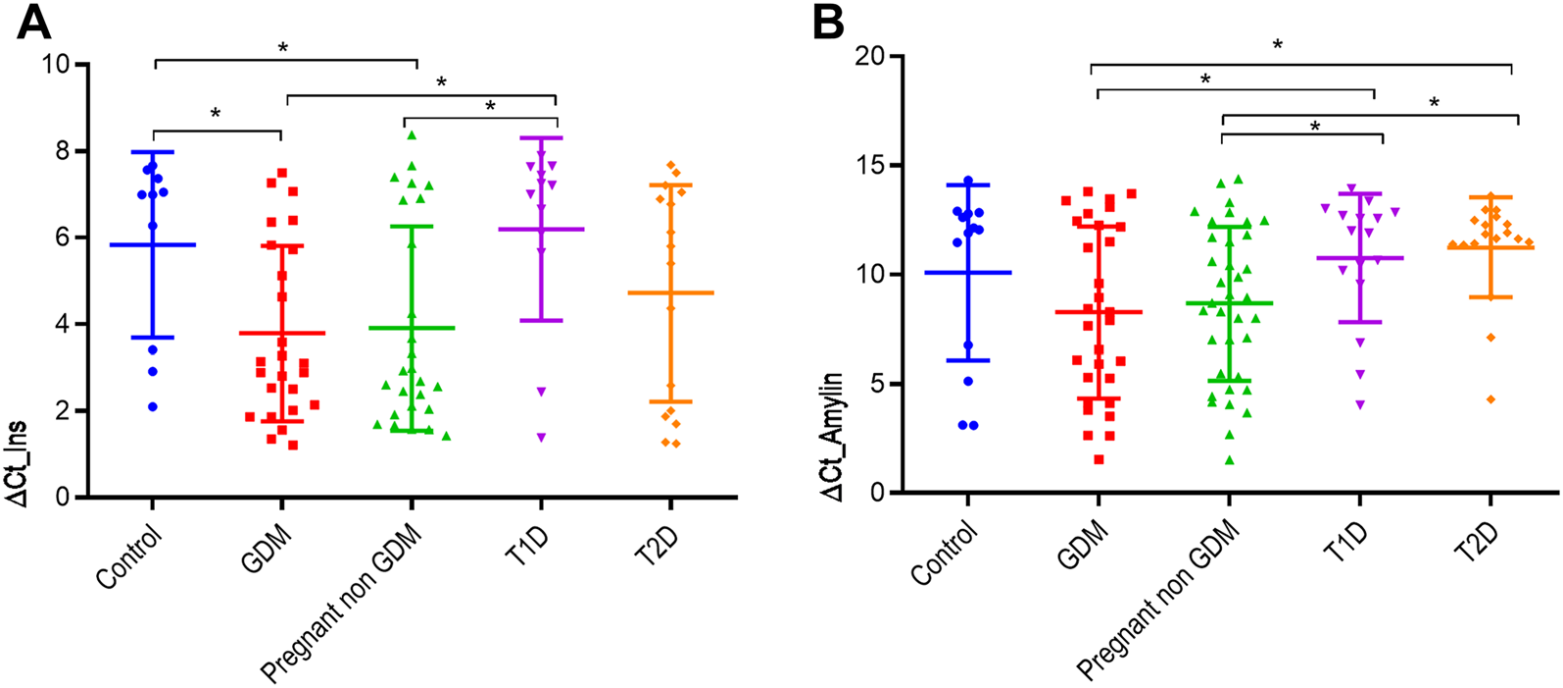
Levels of β cell death markers in the different groups**.** Levels of β cell death markers in healthy controls, GDM and non-GDM pregnant women, Type 1 diabetes (T1D) and Type 2 diabetes (T2D) subjects. **A** ∆Ct for Insulin β-cell death marker. **B** ∆Ct for Amylin β cell death marker. *Statistical significance was assumed at p < 0,05. Data are expressed as mean ± standard deviation. Asterisks indicate statistically significant differences between groups by Duncan test

We observed statistically significant differences between β-cell markers (*INS* and Amylin) levels in the different groups. Curiously, pregnant women (with and without GDM) showed the lowest levels of methylation index, indicating a higher β-cell death for both markers. However, healthy subjects and T1D showed similar levels of methylation index for *INS* and Amylin markers, whereas subjects with T2D presented intermediate levels (Fig. 1**)**. Differences in the INS β-cell marker remained statistically significant even after adjusting for age, weight, and glucose levels (p = 0.035). We did not find significant differences in both indexes of β-cell death among pregnant women with and without GDM. These indexes (INS and Amylin) correlated positively (r = 0.215; p = 0.036).

### Association of β cell death marker with biochemical and anthropometric variables

Due to the different nature of each group of subjects, the analysis was performed stratifying by group.

#### Pregnant women

**Fig. 2:**
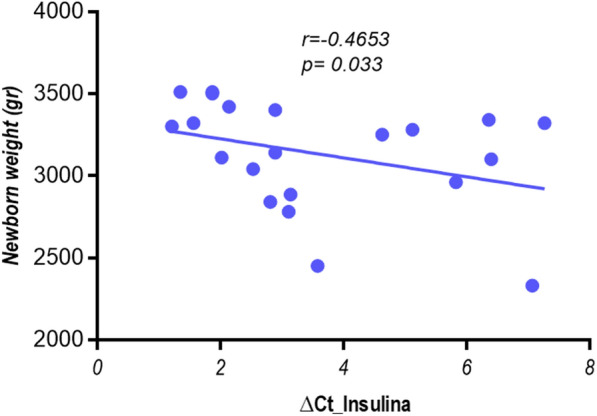
Relationship between newborn weight and INS marker in the GDM group. Correlation analysis between newborn weight and INS β cell death marker in the GDM group by Spearman test. Statistical significance was assumed at p < 0,05. *∆Ct* Ct unmethylated-Ct methylated). Low values indicate a higher proportion of unmethylated DNA, therefore a higher rate of β-cell death

When we analyzed all the pregnant women, no statistically significant associations were found for any variable (age, glucose, weight, insulin, HOMA-IR, newborn’s weight and height) and the β cell death markers. However, when this group of women was analyzed according to their metabolic status (GDM or non- GDM), we observed a negative correlation between *INS* β-cell marker and the newborn weight (r = -0.46; p = 0.033) in those pregnant women with GDM (Fig. 2). So, a larger birth weight was associated with a higher level of β-cell death. Nevertheless, no association was found in pregnant women without GDM.

**Fig. 3:**
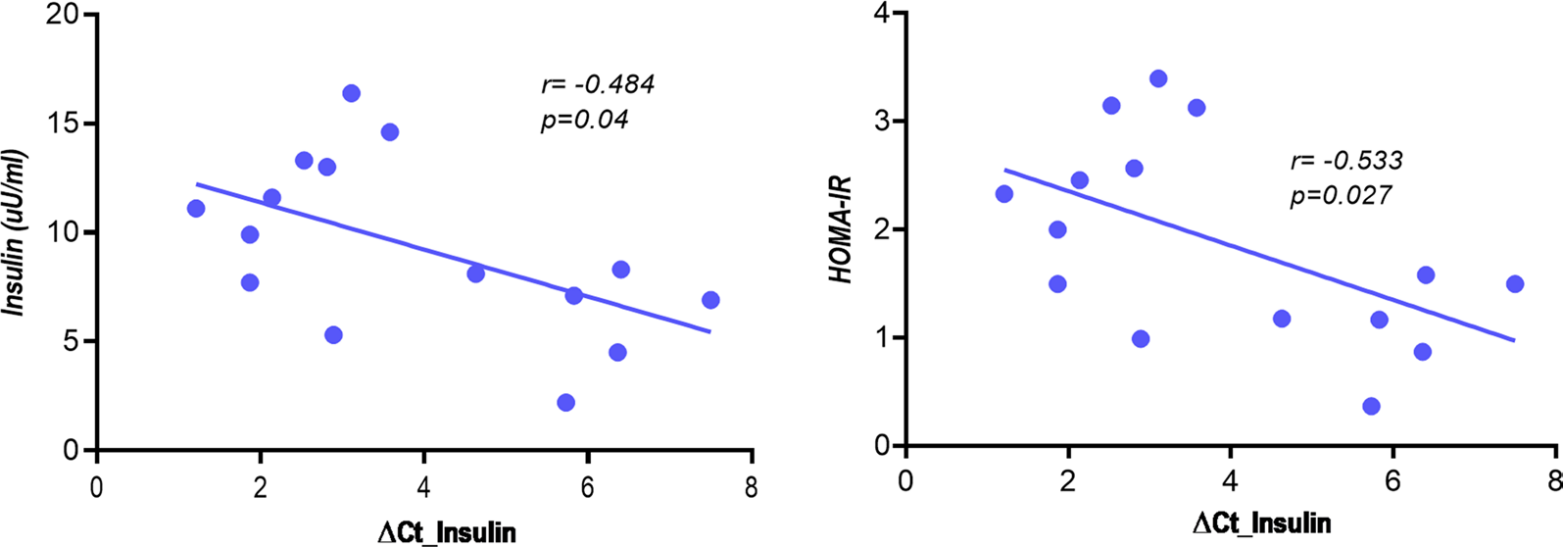
Correlation of insulin parameters and INS β cell death marker. Correlation analysis between serum insulin and HOMA-IR with INS β cell death marker in pregnant women with GDM and HOMA-IR below percentile 75. Spearman test. Statistical significance was assumed at p < 0,05. *∆Ct* Ct unmethylated-Ct methylated). Low values indicate a higher proportion of unmethylated DNA, therefore a higher rate of β-cell death

On the other hand, we analyzed the levels of these β-cell death markers according to the insulin resistance index, HOMA-IR. In this way, women were classified based on the HOMA 75 percentile. In the group of pregnant women with GDM and HOMA-IR < P75, *INS* β-cell marker was negatively associated with insulin levels and HOMA-IR index (Fig. 3) indicating that lower in*s*ulin levels and HOMA-IR are related with reduced β-cell death.

#### Subjects diagnosed as T1DM or T2DM

Subjects who attended for first time to the Diabetes Day Hospital with the clinical onset of diabetes were diagnosed as T1DM (44%) or T2DM (56%). 60% of the T1DM subjects presented GAD autoantibodies, whereas around the 47% had IA2 autoantibodies. We found no significant differences between both β-cell death markers and subjects with T1D or T2D. On the other hand, no association was observed among these markers and C-Peptide levels, or presence of autoantibodies.

## Discussion

This study was designed to analyze the rate of β-cell death in different groups of diabetes mellitus (GDM, T1D and T2D) by two potential biomarkers (INS and Amylin). Our main finding is the detection of β- cell death in pregnant women, both with GDM and non-GDM; and the association with relevant parameters as well as insulin resistance and newborn birth weight in the GDM group.

A large number of studies have been addressed on T1D due to great interest in identifying noninvasive biomarkers that allow us to detect the onset of the disease before the symptoms appear and the β-cell destruction has not been completed yet. Based on the approach of specific methylation pattern from circulating DNA, several studies have reported the presence of β cell-derived cfDNA in individuals at risk of T1D and patients recently diagnosed [10, 11, 22, 24]. However, other authors have shown inconsistent results [25]. In our study, T1D subjects showed the lowest rate of β cell death compared with the other groups. These results are consistent with those recently published by Neiman et al. [25]. These authors showed for the first time the presence of unmethylated Ins cfDNA in T1D subjects [7]. Later, in a more rigorous and exhaustive, they did not observe an increase of β cell-derived cfDNA in autoantibody positive subjects at risk for T1D, individuals with recent onset T1D or those with longstanding disease [25]. These discrepancies could be explained by several facts. On the one hand, the different methodological processes and the confounders variables affecting these assays should be considered: type of sample (serum versus plasma); preanalytical processing (time between collection and centrifugation, double centrifugation); isolation of cfDNA using specific kits, volume of sample and finally, the targeted CpGs sites in INS gene [5, 26, 27]. On the other hand, in our study, the patients who attended to the clinic with a case of hyperglycemia, have probably already suffered the preclinical phase in which the beta cell destruction occurs. So, if T1D is advanced, the number of live beta cells is very low, thus the rate of cell death is also low. The remaining beta cells produce small amount of insulin, what relates to low level of peptide-c. Thus, low level of beta cell markers indicates that there is low number of live beta cells that may undergo death, thereby the rate of beta cell death is the lowest. Maybe this could explain the similar methylation levels in T1D patients and control group. At diagnosis, there is not enough beta cell, and maybe the method is not sensitive enough to detect a signal of β cell destruction in this stage [28]. Even, some authors suggest that autoimmune β cell death does not release such fragments of circulating DNA into the circulation, because they are previously phagocytosed [25]. In any case, our assay did not detect β cell death by these two markers (Ins and Amylin) in recently diagnosed subjects with diabetes.

Regarding to other metabolic phenotypes, to the best of our knowledge, only two studies have explored the presence of this marker in women with gestational diabetes. The first one, performed by Akirav et al. [29], measured levels of unmethylated *Ins* cfDNA in non-pregnant women, pregnant, pregnant with GDM and postpartum without previous diabetes. They did not find an increase of β cell death in GDM compared with the other groups. The other study analyzed the levels of cell free circulating methylated and unmethylated *Ins* DNA in plasma of GDM women followed up to 10 years for the development of T2D. Samples were collected twelve weeks at postpartum [30]. They found that postpartum levels of cfDNA *Ins* marker were significantly higher in those women with a previous GDM who later developed T2D. However, both studies have several limitations and can not be comparable with ours. Samples from Akirav study were obtained from serum and it is known that cfDNA levels in serum appear significantly higher than in plasma due to contamination from genomic DNA (gDNA). Quantification and integrity of cfDNA fragments should be measured. None of these studies used specific kit to isolate cell free DNA to avoid carrying over larger fragments that could indicate genomic contamination. Finally, cfDNA extraction requires of greater volume of sample to obtain enough DNA. Minimum volumes of 1 ml are highly recommended. All these recommendations are being incorporated into the last years according to the literature to guarantee that circulating cell free DNA is properly isolated [26]. One of the main strengths of this study is the rigorous methodology. First, we have processed all the samples following the same protocol and within 4 h post collection. A double centrifugation to minimize the potential for contamination of plasma with cells from the buffy layer were carried out. All the samples were quantified and visualized by the Agilent 2200 TapeStation system to ensure the presence of cfDNA around 200 pb and non gDNA contamination. Moreover, two biomarkers (INS and Amylin) were analyzed for each sample. We have shown that demethylation index of both markers is decreased in pregnant women and there is an association with parameters related with β cell function, as insulin resistance in the GDM group. In addition, among pregnant women with GDM, those with a higher rate of β cell death gave birth newborns with higher birth weight. During pregnancy an increased insulin resistance is produced independently of the metabolic status, to compensate the greater substrate availability required by the fetus. In this period β cell adaptation occurs by increasing mass, number, and glucose-stimulated insulin secretion [31]. This effect is observed in all the pregnant women and could be reflected in a higher β cell death due to a higher turn-over process. In addition, Salazar suggests that the increase in β cell mass occurs in the first half of pregnancy and, when we look at it, we are seeing whether these adaptations have been sufficient to counteract the physiological insulin resistance during pregnancy. Maybe the beta cell death is similar in both groups, but the mass of beta cells that is achieved is lower in those who end up developing GDM and most likely it influences in the development of GDM. This could explain our finding of a higher β cell death in both groups of pregnant women with GDM and non-GDM. Regarding GDM group, we observed an interesting relationship between β cell death and the newborn weight and insulin resistance. This finding is interesting because reinforce what we already know, that GDM pregnant women have newborn with higher birthweight and a worst metabolic profile, but introduce a new variable in this observation, their potential association with beta cell death.

Our data show for the first time that β cell death occurs during pregnancy, and probably this could be more significant in GDM pregnant women, but more studies with larger samples and followed-up over time, are needed.

Lastly, our study highlights the use of the demethylated amylin cfDNA index to detect β cell death. Until date, only one study has reported the use of this measurement as biomarker of β-cell loss[14]. We observed the same trend in the demethylation index of Amylin than the Insulin, although none significant associations with other parameter were found. Amylin index showed a more homogenous pattern among the different groups compared with the Insulin as we can observe in the Fig. 1. So, it is possible that a greater number of samples might be required to detect statistically significant differences with this marker.

This study has some limitations. The main one is the number of samples per group. Additionally, this is an observational study, therefore a follow-up to assess the evolution of these biomarkers along the time, would reinforce these results. Finally, it should be mentioned that cell free DNA from maternal plasma contain a small percentage of fetal DNA. However, only the 10% of the total cfDNA is fetal. So we assume that this amount of fetal DNA is minimal compared to the amount we detected from the mother, considering that our results are not influenced by fetal cfDNA fraction [32, 33].

In conclusion, we investigated the use of two potential biomarkers of β cell death in a group of subjects with different type of diabetes. We observed that both markers (INS and Amylin) presented the same trend in the studied groups. The main finding was the detection of β cell death in pregnant women independently of their metabolic status. This rate of β cell loss was associated with increased insulin resistance and newborn weight in GDM women.

## Supplementary Information

**Additional file 1: Table S1. **Primers sequences and PCR protocols for Insulin and Amylin assays.

## Data Availability

The data sets used during the current study are available from the corresponding author on reasonable request.
