# Supplementary material for: Higher β cell death in pregnant women, measured by DNA methylation patterns of cell-free DNA, compared to new-onset type 1 and type 2 diabetes subjects: a cross-sectional study
Source: Diabetol Metab Syndr. 2023 Jun 1;15:115. doi: 10.1186/s13098-023-01096-9 (PMC10234021; doi:10.1186/s13098-023-01096-9)
Supplement: Supplementary file 1 — Additional file 1: Table S1. Primers sequences and PCR protocols for Insulin and Amylin assays. [file 13098_2023_1096_MOESM1_ESM.docx]

Additional file 1: Table S1- Primers sequences and PCR protocols for Insulin and Amylin assays

| **Gene** | **PCR Type** | **Primer Designation** | **Primer Sequence 5'- 3'** | **Product Lenght (nt)** | **PCR Protocol** |
| --- | --- | --- | --- | --- | --- |
| **Insulin** | Independent methylation PCR | Foward | GGTTTTTTGGGGATTTGATT | 153 | 30 cycles, annealing Tº 58ºC |
|  |  | Reverse | AACTCACCCTACAAATCCTCT |  |  |
|  | Methylation-specific qPCR | Comun Foward | ATTTGGTGGAAGTTTTTTATTTAGTG | 85 | 40 cycles, annealing Tº 61ºC |
|  |  | Methylated specific | ACCCTACAAATCCTCTACCTCCCGACG |  |  |
|  |  | Unmethylated specific | CTACAAATCCTCTACCTCCCAACA |  |  |
| **Amylin** | Independent methylation PCR | Foward | TGTTATTAGTTATTAGGTGGAAAAG | 146 | 45 cycles, annealing Tº 63ºC |
|  |  | Reverse | TCTTACCATATATATTAAATCCCAC |  |  |
|  | Methylation-specific qPCR | Comun Foward | TGTTATTAGTTATTAGGTGGAAAAG | 76 | 40 cycles, annealing Tº 61ºC |
|  |  | Methylated specific | TAAAAAATTTACCAAACGCTACG |  |  |
|  |  | Unmethylated specific | TAAAAAATTTACCAAACACTACA |  |  |

**Protocol Nested-PCR:**

To perform the PCRs, previously we converted with bisulfite (Epitect Bisulfite Kit Qiagen GmbH., Hilden, Germany), the total volume of cfDNA (20 µl).

First PCR: Independet methylation PCR:

SensiFAST SYBER Lo-ROX: 10 µl

Primer forward independent methylation Ins/Amy (10uM): 0.8 µl

Primer reverse independent methylation Ins/Amy (10uM): 0.8 µl

RNAase-free water: 6.4 µl

After the first PCR, we dilute the samples. In the case of insulin, they were diluted 1:100.000, and in the case of amylin they were diluted 1:1.000.000. Then a methylation specific qPCR was performed.

Methylation specifc qPCR:

SensiFAST SYBER Lo-ROX: 10 µl

Primer forward methylation specific Ins/Amy (10uM): 0.8 µl

Primer Reverse methylation specific Ins/Amy (10 uM): 0.8 µl

Molecular biology water: 6.4 µl
